# Whale, Whale, Everywhere: Increasing Abundance of Western South Atlantic Humpback Whales (*Megaptera novaeangliae*) in Their Wintering Grounds

**DOI:** 10.1371/journal.pone.0164596

**Published:** 2016-10-13

**Authors:** Guilherme A. Bortolotto, Daniel Danilewicz, Artur Andriolo, Eduardo R. Secchi, Alexandre N. Zerbini

**Affiliations:** 1 Pós-graduação em Zoologia, Universidade Estadual de Santa Cruz, Ilhéus, BA, 45662–900, Brasil; 2 Instituto Aqualie, Av. Dr. Paulo Japiassu Coelho, 714, Sala 202, Juiz de Fora, MG, 36033–310, Brasil; 3 Sea Mammal Research Unit–SMRU, Scottish Oceans Institute, University of St Andrews, St Andrews, Fife, KY16 8LB, United Kingdom; 4 Grupo de Estudos de Mamíferos Aquáticos do Rio Grande do Sul–GEMARS, Av. Tramandaí, 976, Imbé, RS, 95625–000, Brasil; 5 Laboratório de Ecologia Comportamental e Bioacústica–LABEC, Departamento de Zoologia, Instituto de Ciências Biológicas, Universidade Federal de Juiz de Fora, R. José Lourenço Kelmer, Juiz de Fora, MG, 36036–900, Brasil; 6 Laboratório de Ecologia e Conservação da Megafauna Marinha–EcoMega, Instituto de Oceanografia, Universidade Federal do Rio Grande, Av. Itália, km 8, Rio Grande, RS, 96203–900, Brasil; 7 Marine Mammal Laboratory, Alaska Fisheries Science Center, NOAA Fisheries, 7600 Sand Point Way NE, Seattle, WA, 98115–6349, United States of America; 8 Cascadia Research Collective, 218 ½ 4^th^ Ave W, Olympia, WA, 98501, United States of America; University of California Davis, UNITED STATES

## Abstract

The western South Atlantic (WSA) humpback whale population inhabits the coast of Brazil during the breeding and calving season in winter and spring. This population was depleted to near extinction by whaling in the mid-twentieth century. Despite recent signs of recovery, increasing coastal and offshore development pose potential threats to these animals. Therefore, continuous monitoring is needed to assess population status and support conservation strategies. The aim of this work was to present ship-based line-transect estimates of abundance for humpback whales in their WSA breeding ground and to investigate potential changes in population size. Two cruises surveyed the coast of Brazil during August-September in 2008 and 2012. The area surveyed in 2008 corresponded to the currently recognized population breeding area; effort in 2012 was limited due to unfavorable weather conditions. WSA humpback whale population size in 2008 was estimated at 16,410 (CV = 0.228, 95% CI = 10,563–25,495) animals. In order to compare abundance between 2008 and 2012, estimates for the area between Salvador and Cabo Frio, which were consistently covered in the two years, were computed at 15,332 (CV = 0.243, 95% CI = 9,595–24,500) and 19,429 (CV = 0.101, 95% CI = 15,958–23,654) whales, respectively. The difference in the two estimates represents an increase of 26.7% in whale numbers in a 4-year period. The estimated abundance for 2008 is considered the most robust for the WSA humpback whale population because the ship survey conducted in that year minimized bias from various sources. Results presented here indicate that in 2008, the WSA humpback whale population was at least around 60% of its estimated pre-modern whaling abundance and that it may recover to its pre-exploitation size sooner than previously estimated.

## Introduction

The humpback whale *Megaptera novaeangliae* (Borowski 1781) is found in all major oceans and typically migrates between summer feeding grounds in high latitudes, and winter breeding grounds in tropical and sub-tropical regions [1]. Whaling greatly depleted populations worldwide [2,3], with more than 200,000 whales caught in the Southern Hemisphere alone [4]. After protection warranted by the International Whaling Commission (IWC) in the late 1960s, most populations have shown signs of recovery (e.g. [5–10]). About a decade ago, the species was re-classified by the International Union for Conservation of Nature (IUCN) Red List, from “Vulnerable” to “Least Concern”, due to increase in population sizes [11]. However, the Arabian Sea and the Oceania population remain listed as “Endangered” because of their relatively small abundances and lack of observable recovery [12,13].

The IWC recognizes seven humpback whale breeding stocks in the Southern Hemisphere [14]. Breeding Stock A corresponds to the western South Atlantic (WSA) population, which inhabits the Brazilian coast during the winter breeding season [15] and migrates to feeding grounds near South Georgia and the South Sandwich islands in summer [16,17]. It has been estimated that this population was depleted to less than 4% of its pre-exploitation size by the mid-1950s as a result of extensive whaling activities in their breeding grounds, migratory routes and, primarily, feeding areas [3,4,18,19].

Most humpback whales along the coast of Brazil concentrate on the Abrolhos Bank (16°40’S–19°30’S), where about 80% of the animals are expected to be found every year during the breeding season [15]. Their known regular range during this period, however, comprises the Brazilian continental shelf and shelf break between Natal (5°S) and Cabo Frio (23°S) [15,16] and increasing records of sightings and strandings beyond this range (e.g. [20–24]) may indicate this recovering population is expanding its distribution range in the WSA breeding grounds.

Such a recovery and expansion of the WSA humpback whale population may result in increased conflicts with anthropogenic activities, including ship strikes [24,25], entanglements in fishing gear [26,27], and those related to the oil and gas industry [28]. Evaluation and appropriate management of the potential impacts of these activities on these animals require accurate assessment of population abundance and trends.

Multiple studies have provided abundance estimates for humpback whales off the Brazilian coast (e.g. [15,29–33]). However, most of them did not cover the entire range of the population in the area. Estimates from those studies that did (e.g. [15,33]), were computed from aerial surveys data and are possibly negatively biased because of the lack of appropriate correction factors to account for visibility bias [34]. In this study we aimed to present new abundance estimates for the humpback whale population breeding in the Brazilian coast, using data from line transect ship surveys. Estimates presented here are compared with previous abundance estimates for the species in the area to assess their relative importance. The new estimates are also discussed in the context of the recovery of the WSA humpback whale population after intense commercial exploitation during the 19^th^ and 20^th^ centuries.

## Material and Methods

Two research cruises were conducted aboard the R/V Atlântico Sul (Universidade Federal do Rio Grande, FURG) in 2008 and 2012. Surveys were designed to search for humpback whales using line transect methods [35].

### Ethics Statement

This study was conducted under permits issued by the Brazilian National Council for Scientific and Technological Development (Conselho Nacional de Desenvolvimento Científico e Tecnológico, CNPq; grant #CMC 026/02-028/03) and the Brazilian Institute of the Environment and Renewable Natural Resources (Instituto Brasileiro do Meio Ambiente e dos Recursos Naturais Renováveis, IBAMA, permit #009/02/CMA/IBAMA; process #02001.000085/02-27; ICMBio #11523–1).

### Survey Area and Survey Design

**Fig 1:**
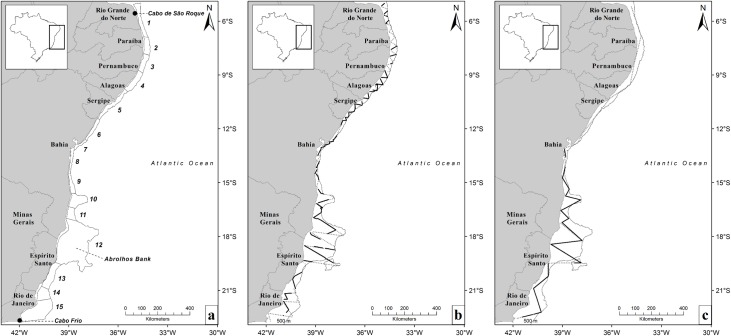
**(a) Survey blocks to estimate the abundance of humpback whales off the Brazilian continental shelf, and (b) survey tracklines in 2008 and (c) in 2012.** On-effort lines (black thick lines) overlap planned lines (grey dashed lines) in **b** and **c.**

The survey area was defined as the coast of Brazil, from Cabo de São Roque (~5°S), Rio Grande do Norte State, to Cabo Frio (~23°S), Rio de Janeiro State, covering the continental shelf from shore to the shelf break, defined in this study as the 500 m isobath limit (Fig 1). Surveys were planned to take place during the seasonal peak of abundance for humpback whales in their breeding grounds off Brazil [36], and were conducted from 25 August to 23 September in 2008, and from 7 August to 3 September in 2012.

Transect lines were zigzag-designed and allocated in 15 strata (i.e. survey blocks; Fig 1) in the 2008 survey. Lines were planned to achieve equal coverage probability [37] and two larger regions were defined according to the amount of relative planned effort: a northern region (blocks 1‒9), and a southern region (blocks 10‒15). The rationale for this division was to focus satellite tagging efforts in the northern region in that year [16,38]. Because of that, planned effort per unit of area in the northern portion was much higher than what was planned for the southern region in 2008.

Tracklines allocation on the 2012 cruise was different from the 2008, because this survey’s focus was deploying tags throughout the study area while still surveying lines in all blocks. Therefore the survey design in 2012 did not follow that of 2008. Additionally, due to unfavorable weather conditions, the survey in 2012 was restricted to the area between Salvador (13°S), in Bahia State, and Cabo Frio, corresponding to the original blocks 8 to 15 (Fig 1). The area of the blocks surveyed in 2008 totaled 123,101 km^2^ and in 2012 totaled 94,295 km^2^.

### Data collection

Tracklines were surveyed in passing mode [39] at an average speed of 17 km h^-1^ and in sea conditions ranging between Beaufort 0 and 6. Searching effort was conducted by a team of nine observers rotating every 30 minutes in three observation positions (port, center and starboard) and one data recording station. After completing a rotation cycle, each researcher rested for at least two hours. The observers were positioned on the flying bridge at 9.5 meters, while the data recorder was located inside the bridge at a computer station. Observers searched for whales independently, with those in port and starboard stations searching from 10° on the opposite side to 90° on their own side. The observer in the center searched between 10° on either side of the trackline. The overlap in the observers’ searching fields was established to minimize the probability of missing animals in the vicinity of the trackline. An additional observer, labelled *tracker*, was located at a 12.6 meters platform (at the crow’s nest of the ship) during the 2012 cruise to assess whether estimation of group size by primary observers was biased. Because effectively no bias was detected [40], the *tracker* data are not considered here. All observers searched for whales with 7x50 reticled binoculars (model Fujinon Mariner XL), which were used for most of the time. For the reminder of the time, searching occurred with naked eye. Observers used angle boards to measure the angle between the sighting and the trackline.

When detections were made, observers provided sighting information (reticle reading, radial angle, species identification and group size) to the data recorder using hand-held VHF radios. Data were immediately logged in a laptop computer equipped with the Wincruz software (R. Holland, SWFSC, NOAA, U.S.A.). Radial distances *r* were calculated from reticle readings using the methods described by Lerczak and Hobbs [41] and perpendicular distances were computed as *x* = *r* ⋅ sin(*θ*), where *θ* is the angle between the group and the trackline. Additionally, group sizes were corrected or confirmed when resightings were possible. When surveying high-density areas, off-effort observers were placed at the flying bridge to help the tracking of groups, minimize double-counting and assist with species identification. Off-effort observers were not involved in searching and did not call in new detections. Environmental data (e.g. sea state, swell height, cloud cover etc.) were recorded and updated in the beginning of every new rotation round or if weather conditions changed.

### Data analysis

Analyses were performed using the beta version of software Distance 7.0 and followed the guidelines suggested in Thomas et al. [42]. Specifically, the following steps were performed: (1) an exploratory analysis for deciding appropriate truncation distances and investigating the need for grouping data in distance intervals (see Buckland et al. [35], page 15); (2) fitting detection probability models and model selection; (3) final analysis and inferences. To model the detection function, right truncation distances were assessed by visually inspecting histograms of perpendicular distances and by fitting preliminary detection probability models. Sightings in low frequencies far from the trackline were excluded. Model selection was conducted in a stepwise approach, using conventional (CDS [35] and multiple covariate distance sampling (MCDS) [43], starting with simple models and including one adjustment term or covariate at a time. Half-normal and hazard rate models were considered as the key function. CDS models included the following adjustment terms: cosine, hermite polynomial and simple polynomial, and MCDS models considered covariates listed in Table 1. Two broader categories of sea conditions were created: calm (Beaufort 0 to 3) and moderate (Beaufort 4 to 6) because sample sizes were insufficient to fit models with individual Beaufort categories. Detection functions for each cruise were estimated with ungrouped data and pooled across geographic strata, but density/abundance, encounter rate and group sizes were computed for each block. Model selection was performed using AIC (Akaike’s Information Criteria). However, if more than one model was well supported by the data (within 2 AIC units), the simplest model, i.e. the one with fewest parameters, was chosen. This resulted in some of the selected models not having the smallest AICs. Empirical variances, coefficients of variation (CVs), 95% confidence intervals (Satterthwaite’s approximation method, [35]) were also estimated in Distance 7.0.

**Table 1:** Candidate covariates tested in the detection function models.

Covariate	Factor/Numeric	Levels
Sea condition	Factor	calm (Beaufort 0–3) and moderate (Beaufort 4–6)
Cue	Factor	splash, body, blow and aerial behavior
Method of detection	Factor	binoculars and naked eye
Group size	Numeric	1–7

Because of the relatively high numbers of unidentified whale sightings in both years, their abundances were also estimated. These estimates were assumed to correspond to humpback whales because sightings of other confirmed large whale species represented less than 1% of all large whale sightings in both cruises. Estimates were summed with those for confirmed humpback whale sightings and combined confidence intervals were computed with the log-based method [44]. Expected group size for unidentified whales was computed from the observed average across all blocks for each cruise.

Because the area surveyed in 2012 was smaller than that of 2008 (blocks 8 to 15, Fig 1), an estimate for the comparable area was also obtained for the 2008 cruise and used to assess potential differences in abundance between those two years. Detection function models used in the estimation of density in the comparable area were the same as those selected to compute abundance for the entire area in that year.

## Results

Completed search effort was 2,337 km in 2008 and 1,683 km in 2012. In total, 416 humpback and 77 unidentified whale groups were sighted in 2008, and 557 humpback and 180 unidentified whale groups in 2012. These numbers were slightly reduced after truncation and are summarized in Table 2. A truncation distance of 4 km was determined for humpbacks in both years and for unidentified whales in 2012, and of 5.5 km for unidentified whales in 2008. Block-specific average group sizes for confirmed humpback whales are also listed in Table 2. Mean group sizes for unidentified large whales were estimated as 1.27 (CV = 0.053) and 1.30 (CV = 0.044), in 2008 and 2012, respectively, after data truncation.

**Table 2:** Effort, sightings and encounter rate per block in 2008 and 2012, after truncation.

		2008										2012									
Block	Area (km^2^	k	L (km)	n HW	n/L HW(CV)		s HW(CV)		n UW	n/L UW(CV)		k	L (km)	n HW	n/L HW(CV)		s HW(CV)		n UW	n/L UW(CV)	
1	2,360	5	96.5	1	0.010	(0.931)	1.00	—	0	0	—	—	—	—	—	—	—	—	—	—	—
2	2,756	2	47.5	2	0.042	(0.313)	1.00	—	0	0	—	—	—	—	—	—	—	—	—	—	—
3	5,348	2	43.5	3	0.069	(0.123)	1.67	(0.400)	1	0.023	(0.543)	—	—	—	—	—	—	—	—	—	—
4	3,713	6	242.7	13	0.054	(0.203)	1.46	(0.166)	2	0.008	(0.597)	—	—	—	—	—	—	—	—	—	—
5	3,802	12	307.1	43	0.140	(0.185)	1.56	(0.074)	8	0.026	(0.581)	—	—	—	—	—	—	—	—	—	—
6	5,145	5	99.6	3	0.030	(0.400)	2.67	(0.250)	2	0.020	(0.717)	—	—	—	—	—	—	—	—	—	—
7	5,681	6	111.6	12	0.108	(0.337)	1.42	(0.136)	2	0.018	(0.682)	—	—	—	—	—	—	—	—	—	—
8	1,400	4	114.5	22	0.192	(0.159)	2.05	(0.113)	2	0.017	(0.750)	2	97.1	9	0.093	(0.405)	1.67	(0.141)	12	0.124	(0.427)
9	2,737	5	89.4	14	0.157	(0.282)	1.93	(0.065)	2	0.022	(0.446)	3	131.9	15	0.114	(0.180)	1.33	(0.094)	11	0.083	(0.629)
10	6,906	3	125.8	12	0.095	(0.331)	1.92	(0.077)	7	0.056	(0.279)	3	219.2	32	0.146	(0.261)	1.41	(0.070)	10	0.046	(0.248)
11	3,533	4	134.8	14	0.104	(0.318)	2.29	(0.191)	5	0.037	(0.298)	3	124.9	20	0.160	(0.146)	1.75	(0.100)	15	0.120	(0.184)
12	49,317	7	605.2	249	0.411	(0.285)	1.96	(0.029)	36	0.059	(0.270)	5	645.0	397	0.616	(0.100)	2.06	(0.029)	96	0.149	(0.052)
13	7,492	3	133.5	5	0.037	(0.958)	1.40	(0.175)	1	0.007	(0.958)	2	180.8	37	0.205	(0.084)	1.68	(0.073)	11	0.061	(0.195)
14	5,379	3	103.1	2	0.019	(0.899)	2.00	(0)	0	0	(0)	2	89.2	10	0.112	(0.411)	1.40	(0.157)	0	0	(0)
15	17,531	2	82.4	5	0.061	(0.486)	2.40	(0.166)	2	0.024	(0.486)	2	195.0	9	0.046	(0.508)	1.44	(0.121)	5	0.026	(0.775)
**Pooled**	**123,101**	**69**	**2337.0**	**400**	**0.171**	**(0.237)**	**1.89**	**(0.024)**	**70**	**0.030**	**(0.211)**	**22**	**1,683.1**	**529**	**0.314**	**(0.247)**	**1.93**	**(0.025)**	**160**	**0.095**	**(0.172)**

k, number of transects; L, realized effort; n, number of sighted groups; HW, humpback whales; CV, coefficient of variation; UW, unidentified whales; n/L, group encounter rate; s, average group size.

**Fig 2:**
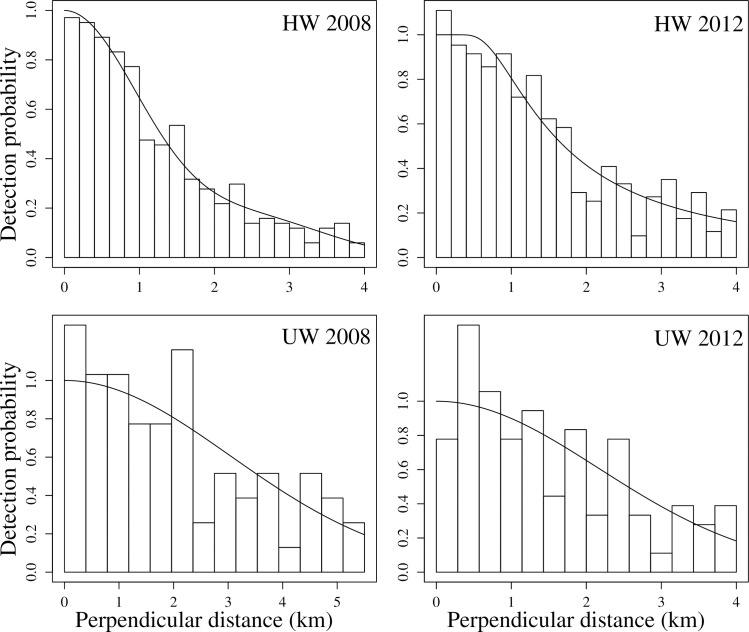
Detection function curves for humpback (HW) and unidentified whales (UW) in 2008 and 2012.

A half-normal model with one cosine adjustment term and a hazard rate model with no extra terms or covariates were chosen, respectively, as the detection probability models for confirmed humpback whale sightings in the 2008 and 2012 cruises. In the case of unidentified whales, models for both 2008 and 2012 were half-normal functions without adjustment terms or covariates. The most supported detection functions for each data type are presented in Table 3. Because the detection probabilities (P; Table 3) were relatively similar within each model set, the criteria used to choose models (i.e. delta AIC < 2 and fewer parameters) works well, and if other models within those in Table 3 were used, little would change on final abundance estimates. The selected models are illustrated in Fig 2.

**Table 3:** Most supported models (delta AIC < 2) for humpback whales (HW) and unidentified whales (UW), in 2008 and 2012.

Year	Species	Key function	Covariate/adjustment	Delta AIC	Par	ESW (km)	P (CV)		GOF–K-S p
**2008**	HW	Half-normal	Cosine	0	2	1.59	0.40	(0.05)	0.9990
**2008**	HW	Hazard Rate	—	1.20	2	1.57	0.40	(0.08)	0.9487
**2008**	HW	Hazard Rate	Group size	1.80	3	1.61	0.40	(0.04)	0.9963
**2008**	UW	Half-normal	—	0	1	3.54	0.64	(0.10)	0.7892
**2008**	UW	Hazard Rate	—	0.72	2	3.08	0.56	(0.22)	0.9769
**2008**	UW	Half-normal	Sea conditions	1.20	2	3.52	0.64	(0.07)	0.8370
**2012**	HW	Half-normal	Cosine	0	3	2.04	0.51	(0.07)	0.9985
**2012**	HW	Hazard Rate	Cosine	0.24	3	1.96	0.49	(0.08)	0.9595
**2012**	HW	Hazard Rate	Sea conditions	1.37	3	2.01	0.50	(0.03)	0.8470
**2012**	HW	Hazard Rate	—	1.47	2	2.06	0.51	(0.06)	0.8648
**2012**	UW	Hazard Rate	—	0	2	2.34	0.58	(0.13)	0.5932
**2012**	UW	Hazard Rate	Sea conditions	0.45	3	2.27	0.57	(0.06)	0.4468
**2012**	UW	Half-normal	—	0.90	1	2.54	0.64	(0.06)	0.5603
**2012**	UW	Half-normal	Method	1.67	2	2.53	0.63	(0.05)	0.7104
**2012**	UW	Hazard Rate	Method	1.69	3	2.38	0.59	(0.05)	0.7692

Chosen models are highlighted in grey. Par, number of parameters; ESW, effective half-strip width; P, probability of detection; CV, coefficient of variation; GOF–K-S p, goodness-of-fit Kolmogorov-Smirnov test probability.

Estimates of density, abundance, CVs and 95% confidence intervals of confirmed humpback whales and unidentified large whales in 2008 are presented in Table 4. The overall estimate for that year when both sets of data were combined was 16,410 (CV = 0.228). Estimates for the surveyed portion of the population range in 2012 (blocks 8‒15) are presented in Table 5. Estimated abundance combining confirmed humpback whales and unidentified large whales in this year was 19,429 (CV = 0.101). The estimate in 2008 for that area was 15,332 (CV = 0.243) and a comparison of these estimates (Table 6) suggest that abundance was 26.7% higher in 2012.

**Table 4:** Humpback and unidentified whales abundance estimates in 2008.

	Humpback whales					Unidentified whales				
Block	D	N	%CV	N 95% CI		D	N	%CV	N 95% CI	
**1**	0.003	8	93.3	1	69	—	—	—	—	—
**2**	0.013	37	31.8	1	1,136	—	—	—	—	—
**3**	0.036	194	42.2	44	847	0.004	22	55.4	0	5,180
**4**	0.025	92	26.9	52	162	0.001	5	60.8	1	23
**5**	0.069	262	20.7	170	403	0.005	18	59.2	5	59
**6**	0.025	130	47.5	43	392	0.004	19	72.6	3	109
**7**	0.048	273	36.8	118	634	0.003	18	69.2	4	89
**8**	0.124	174	20.3	109	277	0.003	4	75.8	1	36
**9**	0.095	261	29.5	123	555	0.004	11	46.0	3	35
**10**	0.058	398	34.4	113	1,399	0.010	69	30.1	25	187
**11**	0.075	265	37.6	107	653	0.007	24	31.8	10	56
**12**	0.254	12,519	29.2	6,303	24,867	0.011	526	29.3	272	1,017
**13**	0.017	124	97.5	5	3,347	0.001	10	96.5	0	302
**14**	0.012	66	90.1	2	1,778	0	0	—	—	—
**15**	0.046	805	51.7	18	35,565	0.004	76	49.9	1	9,003
**Pooled**	**0.127**	**15,607**	**23.9**	**8,950**	**27,215**	**0.007**	**803**	**21.9**	**503**	**1,279**

D, density of animals; N, abundance of animals; CV, coefficient of variation; CI, confidence interval.

**Table 5:** Humpback and unidentified whales abundance estimates in 2012.

	Humpback whales					Unidentified whales				
Block	D	N	%CV	N 95% CI		D	N	%CV	N 95% CI	
**8**	0.038	53	43.4	2	1,130	0.032	44	43.5	0	4,194
**9**	0.037	101	21.4	56	183	0.021	58	63.5	5	665
**10**	0.050	344	27.8	132	898	0.012	81	26.1	31	206
**11**	0.068	241	18.9	150	385	0.031	109	20.0	56	210
**12**	0.308	15,177	12.9	11,509	20,013	0.038	1,877	9.4	1,551	2,272
**13**	0.083	624	12.9	451	864	0.016	117	21.0	27	507
**14**	0.038	205	44.5	11	3,832	—	—	—	—	—
**15**	0.016	284	52.7	3	29,323	0.007	115	77.9	0	486,360
**Pooled**	**0.181**	**17,028**	**11.4**	**13,245**	**21,892**	**0.025**	**2,401**	**9.9**	**1,965**	**2,933**

See Table 4 for legend.

**Table 6:** Combined estimates for humpback and unidentified large whales.

Pooled estimate	D	N	%CV	N 95% CI	
**2008 all blocks**	0.133	16,410	22.8	10,563	25,495
**2008 blocks 8‒15**	0.163	15,332	24.3	9,595	24,500
**2012 blocks 8‒15**	0.206	19,429	10.1	15,958	23,654

See Table 4 for legend.

Block-specific estimates show that humpback whales distribution was not uniform across the surveyed area. Block 12 comprised Abrolhos Bank and had the highest density, including 85% and 88% of the total estimated abundance for 2008 and 2012, respectively. Some other regions also had relatively higher concentration of whales, such as the central coast of Bahia (blocks 8 and 9) in 2008, while areas further north (blocks 1 and 2) had very low densities. In 2012, the highest concentrations outside the Abrolhos Bank were found in adjacent areas, in blocks 10, 11 and 13.

## Discussion

### Abundance

In this study, abundance estimates were computed for 2008 and 2012 using line transect ship surveys data. The estimate obtained from the 2008 cruise data represents the best estimate for the size of the humpback whale population wintering off Brazil, because that survey covered the majority of the species range in their breeding grounds. That estimate suggests that there were nearly 16,400 whales off the Brazilian coast in 2008. The 2012 estimate, while higher, did not cover the total population range and therefore does not reflect its size in the wintering ground off Brazil.

Over the past 20 years, multiple surveys have been conducted in this wintering ground to estimate abundance of the western South Atlantic humpback whales (see a summary in Table 7). Abundances estimated in each study should be interpreted taking into consideration their specific constraints. Most important, the majority of these surveys, including those from mark-recapture techniques, are likely not representative of the size of the population, since they covered a fraction (sometimes a small fraction) of the range of humpback whales off Brazil. Exceptions include, in addition to the 2008 cruise presented here, studies 8 and 9 in Table 7, which corresponded to aerial surveys conducted, respectively, in 2005 [15] and in 2008 [33]. In fact, the estimate computed from the 2005 aerial survey [15] was considered the most representative of the population size by the International Whaling Commission and used in the most recent assessment of the WSA humpback whale population [3].

**Table 7:** Previous studies presenting data on abundance and group sizes for humpback whales off the coast of Brazil.

Survey	Platform	Region	Area (km^2^)	Year	N (%CV)	N 95% CI	GS (%CV)	Method	Reference
**1**	Vessel	Abrolhos bank	—	1995	1,634 (9.5)	1,379–1,887	—	Mark-recapture	[29]
**2**	Vessel	Northern portion of the Abrolhos bank	—	2000	3,871	2,795–5,542	—	Mark-recapture	[30]
**3**	Vessel	Rio Grande do Norte to Sergipe	20,040	2000	628 (33.5)	327–1,157	1.95 (8.8)	Line transect	[31]
**4**	Airplane	Bahia and Espirito Santo	86,225	2001	2,229 (31.3)	1,201–4,137	1.52 (4.6)	Line transect	[32]
**5**	Airplane	Bahia and Espirito Santo	81,103	2002	3,396 (14.2)	2,562–4,501	1.52	Line transect	[15]
**6**	Airplane	Bahia and Espirito Santo	81,103	2003	3,661 (13.1)	2,819–4,756	1.79	Line transect	[15]
**7**	Airplane	Bahia and Espirito Santo	81,103	2004	5,353 (12.8)	4,146–6,913	1.57	Line transect	[15]
**8**	Airplane	Rio Grande do Norte to Rio de Janeiro^a^	160,004	2005	6,404 (11.6)	5,084–8,068	1.63 (1.7)	Line transect	[15]
**9**	Airplane	Rio Grande do Norte to Rio de Janeiro^a^	160,004	2008	9,330 (28.1)	4,857–20,300	1.59 (6.9)	Line transect	[33]
**10**	Vessel	Rio Grande do Norte to Rio de Janeiro^a^	123,101	2008	16,410 (22.8)	10,563–25,495	1.89 (2.5)	Line transect	Present study
**11**	Airplane	Sergipe to Rio de Janeiro	130,546	2011	14,315 (28.7)	8,257–24,818	1.679 (2.2)	Line transect	[45]
**12**	Vessel	Bahia and Espirito Santo(coastal shipping route)	7,207	2011	382 (10.4)	303–454	1.81 (3.4)	Line transect	[25]
**13**	Vessel	Bahia to Rio de Janeiro	94,295	2012	19,429 (10.1)	15,958–23,654	1.93 (2.5)	Line transect	Present study

N = abundance; CV = coefficient of variation; CI = confidence interval; GS = mean group size.

^a^Humpback whales breeding range off the Brazilian coast. The larger survey area in studies 8 and 9 is explained by the inclusion of regions beyond the shelf-break in the southern portion of the area.

A comparison of ship and aerial surveys shows that the estimates of abundance computed from different survey platforms are substantially different, when equivalent time periods are considered. For example, the point estimate for the abundance from the 2008 ship survey in this study (16,410 whales) is 76% greater than that of the aerial survey conducted in the same year and approximately in the same area (9,330 whales; [33]).

Assuming that the ship-based estimate presented here is accurate, the aerial is then biased low, which can be explained by a number of reasons. First, aerial surveys conducted using passing mode tend to underestimate group size. This is clearly visible in Table 7, where mean group sizes estimated from aircrafts ranged from 1.52 to 1.79 while estimates from vessel surveys ranged from 1.81 to 1.95. A comparison of estimates of mean group sizes from the 2008 aerial (1.59 individuals/group, CV = 0.069) and ship surveys (1.89 individuals/group, CV = 0.025) reveal that the former is 19% lower and that the two estimates are significantly different (t = 45.24, df = 391, p < 0.001). Second, aerial surveys rarely meet the line transect sampling assumption that detection on the trackline is certain and require the development of correction factors to account for availability and perception biases (*sensu* [34]) that may cause underestimation in abundance [46]. The aerial surveys conducted off Brazil for humpback whales were only corrected for availability bias. Although perception bias is typically assumed to be low in aerial surveys for large whales, if not accounted for, may be a considerable source of abundance underestimation [47]. Additionally, the inclusion of a correction factor adds another source of uncertainty in the estimates [46].

For the aerial survey conducted off Brazil in 2008 (study 9, Table 7), perception bias could have been magnified because of changes in the survey methodology. Previous surveys (2001‒2005) were carried out at altitudes of 500 feet (152.4 m) [15,32], while the 2008 one was carried out at an altitude of 1000 feet (304.8 m) [33]. This change in altitude could have led to an increased number of missed sightings because whale groups become a smaller target to observers trained to search for whales with the aircraft flying closer to the ground.

The correction factor for availability bias computed by Andriolo et al. [32] and extended or adapted to subsequent abundance estimates obtained through aerial surveys off Brazil (studies 4–9, Table 7) was calculated based on surface and dive times taken from a shore-based observation platform in the Abrolhos Archipelago [32]. Because animals in shallow waters tend to spend more time at the surface when compared to animals at deeper waters, their sample is likely to be biased, and the mean surface time when extrapolated for the population is probably overestimated. In addition mother-calf groups were more represented (77%) on their sample. This class of group is also likely to spend more time near the surface than, for example, solitary animals or groups of mother, calf and one escort [48–50]. These issues may lead to an overestimation of availability bias, and when correction factors are applied to data collect in areas including deeper water habitats, resultant abundances will be underestimated. The same problem applies when the correction is applied to groups without calves, which correspond to the majority of the sightings detected from those aerial surveys. The proportion of groups with calves ranged between only 4% and 13.7% in Andriolo et al. [15] and in Wedekin [33], respectively. A positive bias in the correction factor, could partly explain the much lower point estimate computed from the aerial survey in 2008 in comparison to the one presented here for the same year. On the other hand, the use of surface and dive times taken from an observation point on land may lead to overestimation of abundances if correction factors for availability bias are underestimated. Cetaceans are expected to be seen from airplanes not only when they are at the surface, but in a certain depth range [47,51]. Therefore the use of dive and surface times collected from a shore-based platform to correct for availability bias in aerial surveys often leads to underestimation of the availability-related correction factor [52], with consequential positive bias in the estimates of abundance.

Another possibility to consider is that the aerial survey estimate is accurate and the 2008 ship survey is biased high. Overestimation of abundance could result from a number of reasons, but all are thought not to be a significant problem in the ship survey reported here. First, if sightings of unidentified whales, which estimates were combined with those of confirmed humpback whales, were in fact from other species, the estimate provided here would be biased high. However, no other large whales have been observed in large numbers in the survey area. For example, sightings of southern right whales (*Eubalaena australis*) had been occasionally reported for other years [53] and are known to be rare in the area. In fact, during the 2008 and 2010 surveys only three sightings of this species were identified. Additionally, the estimated abundance of unidentified whales computed for 2008 was relatively small when compared to that of confirmed humpback whales. It represented nearly 5% of the overall abundance, indicating that any positive bias resulting from assuming that these whales were humpback whales would be small. Overestimation could also be possible if the whales were moving or migrating in the same direction the ship was traveling during the survey, which could be conceivable, especially for whales inhabiting the northern range of the survey area, and/or in the end of the season, when whales begin to migrate to their feeding grounds. This problem is believed to be minimized by conducting the surveys during the peak of abundance of whales in their wintering grounds, before migration started. Martins et al. [36] showed that humpback whale density in their breeding grounds off Brazil reached its maximum in August–September and that abundance of some reproductive classes (e.g. solitary animals or pairs) did not decrease until October. In addition, an examination of the movement patterns of whales satellite tagged during the 2008 cruise did not show evidence that whales were moving consistently in the same direction of the survey (*Instituto Aqualie*, unpublished data).

Other sources of positive bias include the potential for double counting, which could occur in areas of high density where distinguishing new groups from those that had already been recorded could be difficult [54]. In the present study, measures to minimize double counting were taken by placing additional observers in the flying bridge to help primary observers to track sightings that had already been called in. Finally, overestimation of abundance could come from surveying high-density areas and extrapolating abundance to low-density regions [35]. However, to reduce this potential issue, the stratified survey design with tracklines allocated in such a way that equal coverage probability was attended should have reduced the chances of having this potential issue. Despite that, survey conditions were such that realized effort did not match proposed effort in some areas. Because realized lines are relatively well distributed in the survey area, there is no reason to believe the present estimates of abundance would be biased. Nonetheless, the application of survey methods that could accommodate uneven coverage (e.g. model-based approaches; [55,56]) would be recommended to assess whether improvements in the estimates provided here could be made. As the number of lines per block implemented in the present surveys (Table 2) were relatively low to adequately estimate variances [35], model-based methods could also be useful to improve precision if the spatial covariates to be used can explain a substantial portion of the density variation across the survey area.

Although the differences between the WSA humpback whale abundance estimates in 2008 from the aerial and the ship surveys (*difference* = 7,110, CV = 0.641) can be justified in many ways, their confidence intervals overlap. However, numbers are undoubtedly different, with the point estimate of the former representing nearly half of the latter. This should be confirmed by a statistical test (z-test), which would require the degrees of freedom of both estimates. While both are subject to potential sources of bias, we consider the ship survey reported here to be robust and more representative of the size of the population wintering in coastal waters off Brazil. This is justified by the more accurate estimates of group sizes and because the probability of missing groups on the trackline is substantially reduced when surveys of marine mammals are conducted from a slow surface platform. In fact, studies conducted to estimate correction factors for whales that are missed on the trackline during shipboard surveys suggested that detection of species with visible bodies and conspicuous blows, such as humpback whales, are close to 100% [57,58].

Even though we consider the abundance estimate computed from the 2008 shipboard survey the most representative for humpback whales wintering along the coast of Brazil, this estimate probably does not correspond the total population size. This occurs because areas along the northern coast of Brazil, north and/or west of 5°S, are used by an unknown, possibly small, number of humpback whales [21,22] and were not surveyed. In addition, the species has also been seen in oceanic islands such as the Fernando de Noronha and the Trindade and Martin Vaz seamounts and archipelagos [23,59,60], which indicates that their wintering range in Brazil may also extend to oceanic islands, far from the coast. Moreover, due to the seasonal and segregated nature of the species migratory cycle [61], not all animals are expected to be in the breeding grounds during the peak of the season. It is possible that some whales may have already left the wintering habitat towards their feeding grounds and others may have not yet arrived when the survey was conducted. Finally, some humpback whales are expected to not migrate in the winter [62] and individuals that stayed in the feeding grounds those years were also not sampled during the surveys. For these reasons, while the estimate for the 2008 ship survey presented here is the one that best approximate the total population size, it is likely biased low.

Regarding the approach of combining the estimated abundances for humpback whales and unidentified large whales adopted here, an alternative would be to consider the sightings from both as being of humpback whales and estimate the distance sampling parameters (i.e. detection probability, group size etc.) from combined detections. However, the present approach aims to clearly separate the contributions of abundance and variance estimates of humpback and unidentified whale sightings on the final (combined) abundance estimates, because of known biases on the unidentified whale data. For example, mean group sizes of unidentified whales are expected to be biased low because the same factors that prevented species identification may be responsible for inaccuracy on group size estimates (e.g. distance to detection and visibility conditions).

### Population increase and status

Abundance estimates provided here for comparable areas indicate that the number of humpback whales wintering off Brazil increased nearly 27% between 2008 and 2012. Because these estimates represent the majority of the population (i.e. abundance in blocks 8 to 15 in 2008 corresponded to 93% of the overall abundance) it is possible that the increase observed between these two years corresponds to the growth of the whole population from 2008 to 2012. Such an increase implies an average annual population growth rate of 6.1%, which is consistent with the point estimate of the rate of increase of 7.4%/year computed from sightings data for WSA humpback whales in the 1990s [10].

The estimates provided above also suggest that the recovery of this population is more optimistic than previously thought. Zerbini et al. [3] presented an assessment of the status of this population and estimated that their pre-exploitation abundance corresponded to nearly 25,000 whales. According to that study, the WSA humpback whale population size in 2006 corresponded to 26–32% of the pre-exploitation size, and that the projected population in 2020 would have recovered to 60% of the pre-whaling abundance. The ship-based estimate in 2008 corresponds to around 65% of the pre-exploitation abundance, indicating the population recovery in the early 2000s was greater than that estimated by Zerbini et al. [3]. Such difference occurred because the assessment model used by the authors assumed the population size at that time (2005) to be the one presented by Andriolo et al. [15], from an aerial survey, which was much lower (N = 6,404, CV = 0.116, 95% CI = 5,084–8,068). If that abundance estimate is biased low due to the reasons discussed above, the recovery of the WSA population estimated by Zerbini et al. [3] was underestimated. The new abundance estimate provided here calls for an update in the assessment of this population.

The conservation status of WSA humpback whales is relatively optimistic when compared to other baleen whale populations around the world [63]. Because the consequences of such an increase in whale numbers on the possible interactions between animals and human activities in the area remain to be seen, monitoring and management actions are still needed. Major concerns arise from the increasing interest in oil and gas extraction in the region, which is set to expand in the near future according to Brazilian National Agency of Petroleum, Natural Gas and Biofuels [64]. For example, acoustical disturbance from seismic surveys are likely to cause behavioral changes that may affect negatively the reproductive success of large whales [65]. We strongly recommend that future studies and assessments for this population consider information on its distribution and potential impacts from human activities.
